# Association between early postoperative hypoalbuminaemia and outcome after orthotopic heart transplantation

**DOI:** 10.1093/icvts/ivae012

**Published:** 2024-01-16

**Authors:** René M’Pembele, Sebastian Roth, Freya Jenkins, Vincent Hettlich, Anthony Nucaro, Alexandra Stroda, Theresa Tenge, Amin Polzin, Bedri Ramadani, Giovanna Lurati Buse, Hug Aubin, Artur Lichtenberg, Ragnar Huhn, Udo Boeken

**Affiliations:** Department of Anesthesiology, Medical Faculty and University Hospital Duesseldorf, Heinrich-Heine-University, Duesseldorf, Germany; Department of Anesthesiology, Medical Faculty and University Hospital Duesseldorf, Heinrich-Heine-University, Duesseldorf, Germany; Department of Cardiac Surgery, Medical Faculty and University Hospital Duesseldorf, Heinrich-Heine-University, Duesseldorf, Germany; Department of Cardiac Surgery, Medical Faculty and University Hospital Duesseldorf, Heinrich-Heine-University, Duesseldorf, Germany; Department of Anesthesiology, Medical Faculty and University Hospital Duesseldorf, Heinrich-Heine-University, Duesseldorf, Germany; Department of Anesthesiology, Medical Faculty and University Hospital Duesseldorf, Heinrich-Heine-University, Duesseldorf, Germany; Department of Anesthesiology, Medical Faculty and University Hospital Duesseldorf, Heinrich-Heine-University, Duesseldorf, Germany; Department of Cardiology, Pulmonology and Vascular Medicine, Medical Faculty and University Hospital Duesseldorf, Heinrich-Heine-University, Duesseldorf, Germany; Department of Cardiac Surgery, Medical Faculty and University Hospital Duesseldorf, Heinrich-Heine-University, Duesseldorf, Germany; Department of Anesthesiology, Medical Faculty and University Hospital Duesseldorf, Heinrich-Heine-University, Duesseldorf, Germany; Department of Cardiac Surgery, Medical Faculty and University Hospital Duesseldorf, Heinrich-Heine-University, Duesseldorf, Germany; Department of Cardiac Surgery, Medical Faculty and University Hospital Duesseldorf, Heinrich-Heine-University, Duesseldorf, Germany; Department of Anesthesiology, Kerckhoff Heart and Lung Center, Bad Nauheim, Germany; Department of Anesthesiology, Amsterdam University Medical Center (AUMC), Location AMC, Amsterdam, Netherlands; Department of Cardiology, Pulmonology and Vascular Medicine, Medical Faculty and University Hospital Duesseldorf, Heinrich-Heine-University, Duesseldorf, Germany

**Keywords:** cardiac surgery, biomarkers, prognosis, risk stratification, liver function

## Abstract

**OBJECTIVES:**

In patients undergoing heart transplantation (HTX), preoperative liver impairment and consecutive hypoalbuminaemia are associated with increased mortality. The role of early postoperative hypoalbuminaemia after HTX is unclear. This study investigated the association between early postoperative hypoalbuminaemia and 1-year mortality as well as ‘days alive and out of hospital’ (DAOH) after HTX.

**METHODS:**

This retrospective cohort study included patients who underwent HTX at the University Hospital Duesseldorf, Germany, between 2010 and 2022. The main exposure was serum albumin concentration at intensive care unit (ICU) arrival. The primary endpoints were mortality and DAOH within 1 year after surgery. Receiver operating characteristic (ROC) curve analysis was performed and logistic and quantile regression models with adjustment for 13 a priori defined clinical risk factors were conducted.

**RESULTS:**

Out of 241 patients screened, 229 were included in the analysis (mean age 55 ± 11 years, 73% male). ROC analysis showed moderate discrimination for 1-year mortality by postoperative serum albumin after HTX [AUC = 0.74; 95% confidence interval (CI): 0.66–0.83]. The cutoff for serum albumin at ICU arrival was 3.0 g/dl. According to multivariate logistic and quantile regression, there were independent associations between hypoalbuminaemia and mortality/DAOH [odds ratio of 4.76 (95% CI: 1.94–11.67) and regression coefficient of −46.97 (95% CI: −83.81 to −10.13)].

**CONCLUSIONS:**

Postoperative hypoalbuminaemia <3.0 g/dl is associated with 1-year mortality and reduced DAOH after HTX and therefore might be used for early postoperative risk re-assessment in clinical practice.

**Figure:**
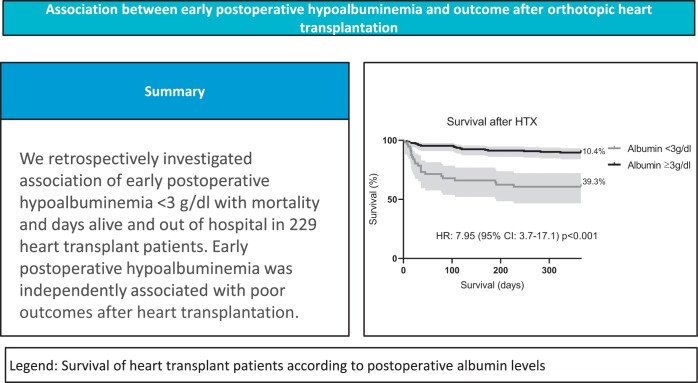

## INTRODUCTION

Postoperative hypoalbuminaemia is frequent after cardiac surgery with the use of cardiopulmonary bypass (CPB) and can result from intraoperative blood loss, dilution, increased inflammatory response or postoperative capillary leak amongst other reasons [1]. Recent studies showed an association of postoperative hypoalbuminaemia with poor short- and long-term survival in patients undergoing cardiac surgery with and without CPB [2]. In patients undergoing heart transplantation (HTX), preoperative risk assessment is crucial to identify patients with poor prognosis. In this context, preoperative liver impairment was identified as a factor which is associated with increased mortality after HTX. Therefore, biomarkers of liver impairment are included in risk prediction tools like the Index for Mortality Prediction After Cardiac Transplantation (IMPACT) score [3, 4]. Recently, the use of the model for end-stage liver disease (MELD) score was proposed for preoperative risk assessment in heart transplant patients [5]. Preoperative serum albumin is another biomarker that is decreased when liver function is impaired. Previous research showed that low preoperative albumin levels as the sole marker or when added to the MELD score were a strong predictor of poor outcome in HTX and heart failure patients [6–9]. However, the role of early postoperative hypoalbuminaemia in risk re-assessment and prognosis after HTX is unclear. Therefore, the aim of this study was to investigate the association between early postoperative hypoalbuminaemia and 1-year mortality as well as ‘days alive and out of hospital’ (DAOH) as a patient-centred outcome after HTX. Furthermore, the additional predictive value of postoperative hypoalbuminaemia for 1-year mortality was assessed as compared to the postoperative MELD score, as a measure for early postoperative liver dysfunction, and the preoperative IMPACT score.

## MATERIALS AND METHODS

### Ethics statement

This analysis was conducted as a retrospective single-centre cohort study. Approval was obtained from the institutional review board of the University of Duesseldorf (reference number: 4567). The extracted data for this analysis were available in the local prospective HTX database. All patients included in this database had given written informed consent to be enrolled. Reporting of the results follows STROBE guidelines [10].

### Patient population and inclusion criteria

Consecutive adult patients (≥18 years) undergoing HTX at a tertiary care University Hospital in Duesseldorf, Germany, from September 2010 to April 2022 with completed 1-year follow-up were screened for inclusion. Patients with missing data regarding the primary endpoints or postoperative serum albumin measurements were excluded from the analysis.

### Perioperative fluid and transfusion management

Perioperative fluid and transfusion management of HTX patients is highly standardized in our institution and guided by continuous haemodynamic monitoring. Intraoperative blood management during and post CPB targets haemoglobin levels around 10 g/dl. Serum albumin levels of 2.0 g/dl or below are used as a threshold for albumin substitution. Albumin substitution is considered at serum albumin levels below 2.5 g/dl if the patient shows signs of peripheral oedema.

### Serum albumin measurements

Main exposure was the first postoperative serum albumin concentration measured in g/dl within the first 12 h at ICU. Albumin values were determined by the local central laboratory.

### IMPACT score calculation

The risk index for IMPACT was calculated for each patient as described previously [3, 4, 11]. It assigns varying points for the variables age, serum bilirubin, creatinine clearance, dialysis, sex, heart failure aetiology, preoperative infection, race, circulatory support and type of ventricular assist device and was calculated for a previously published analysis [11]. Data were received by the prospective local database that stored manually extracted information from electronic clinical charts by trained personnel.

### MELD score calculation

The MELD score was calculated for each patient as described previously using the following formula: 10 × [0.957 × Ln(Creatinine) + 0.378 × Ln(total bilirubin) + 1.12 × Ln(international normalized ratio) + 0.643] [12]. For calculation, immediate postoperative values of these biomarkers were used.

### Outcomes

The primary endpoint was all-cause mortality during the first year after surgery. The secondary endpoint was the number of DAOH within the first year after HTX. DAOH were calculated by subtraction of all days spent in the hospital from 365 days. In case of death, the days the patient did not survive were added to the time spent in the hospital which was then subtracted from 365 days [13, 14]. DAOH is a more patient-centred outcome as it includes mortality, length of hospital stay and hospital readmissions and is known for its correlation with measures of quality of life [11].

### Statistical analysis

Statistical software used for the present analysis were IBM SPSS software version 25.0 (Armonk, NY, USA), GraphPad Prism version 8.02 (La Jolla, CA, USA) and MedCalc Statistical Software version 20.114 (MedCalc Software Ltd, Ostend, Belgium). Descriptive statistics are presented as number (*n*) with corresponding percentages (%) in brackets for categorical variables and as mean ± standard deviation (SD) for continuous variables. Fisher’s exact test or unpaired *t*-tests were used to compare continuous or dichotomous variables between groups. To evaluate the prognostic value of postoperative albumin receiver operating characteristic curve (ROC) analysis was conducted (dependent variable: 1-year mortality). The Youden index was used to determine a cutoff value for albumin level. Kaplan–Meier curves were conducted for survival analysis depending on albumin cutoff value. Univariate logistic regression was conducted for postoperative albumin level and 1-year mortality. In a multivariate logistic regression model, odds ratios (ORs) were adjusted for clinical risk factors from baseline characteristics that might be associated with 1-year mortality. To evaluate if postoperative albumin could improve risk stratification of mortality prediction models, the net reclassification improvement (NRI) and the net absolute reclassification improvement (NARI) of two mortality prediction models adding postoperative albumin level to either MELD score or IMPACT score were calculated as previously performed [11]. Discrimination of these models with and without the addition of albumin was assessed (ROC-AUC) and compared using the Delong test. DAOH of patients with albumin values higher and lower than Youden index derived cutoff were compared in univariate analysis using Mann–Whitney *U* test. In a multivariate quantile regression model of the lowest DAOH centile, association of continuous postoperative albumin values with DAOH was adjusted for clinical risk factors from baseline characteristics. For all statistical tests, a *P*-value of <0.05 was considered significant. Similar methods were used in previous publications [11].

## RESULTS

### Study cohort and characteristics

A total of 241 patients underwent HTX from 2010 to 2022 at the University Hospital of Duesseldorf and completed 1-year follow-up. Based on the inclusion and exclusion criteria 12 patients had to be excluded and 229 patients were included in the analysis. Mean age of the study group was 55 ± 11 years. Overall 1-year mortality was 17.4% (40 patients) and 30-day mortality was 7.8% (18 patients). Median DAOH were 299 (230–322) at 1 year after HTX. Detailed patient characteristics are presented in Table 1 (Supplementary Table S1, Figure S1).

**Table 1:** Patient characteristics by postoperative albumin levels

	Albumin ≥3 g/dl (*N* = 173)	Albumin <3 g/dl (*N* = 56)	*P*-value
Preoperative recipient characteristics			
Male sex	129 (74.6)	38 (67.9)	0.387
Age (years)	55.1 ± 10.8	55.3 ± 11	0.904
BMI (kg/m^2^)	25.8 ± 4.5	25.4 ± 4.7	0.614
Smoker	45 (26.2)	12 (21.4)	0.594
Diabetes	32 (18.7)	18 (32.1)	**0.042**
Arterial hypertension	98 (57)	32 (57.1)	>0.999
Pulmonary hypertension	14 (8.1)	7 (12.5)	0.424
Prior cardiothoracic surgery	101 (58.4)	44 (78.6)	**0.007**
LVAD	78 (45.1)	37 (66.1)	**0.009**
ICM	73 (42.4)	25 (44.6)	0.877
DCM	81 (47.1)	28 (50)	0.759
ARVC	7 (4.1)	1 (1.8)	0.683
RCM	0 (0)	1 (1.8)	0.246
HCM	3 (1.7)	1 (1.8)	>0.999
Myocarditis	2 (1.2)	0 (0)	>0.999
Preoperative dialysis	10 (5.8)	2 (3.7)	0.736
IMPACT score	8.1 ± 4.7	9.4 ± 4.1	0.096
Albumin (g/dl)	3.9 ± 0.7	3.7 ± 0.7	0.185
MELD score	14.3 ± 7.6	13.7 ± 6.5	0.652
Donor characteristics			
Male sex	98 (56.6)	28 (50)	0.441
Age (years)	43.1 ± 12.2	44.3 ± 12.4	0.526
BMI (kg/m²)	26.1 ± 4.9	26.0 ± 3.7	0.945
Cardiopulmonary resuscitation	53 (30.6)	15 (26.8)	0.618
Intraoperative characteristics (min)			
Duration of surgery	401 ± 95	490 ± 148	**<0.001**
Duration of CPB	240 ± 57	294 ± 94	**<0.001**
Total ischaemia time	212 ± 48	220 ± 51	0.330
PRBC (l)	2.9 ± 2.4	4.9 ± 3.2	**<0.001**
Platelets (l)	1.0 ± 0.8	1.5 ± 1.1	**0.001**
FFP (l)	1.5 ± 1.6	1.9 ± 2.3	0.218
Postoperative laboratory values			
Creatinine (mg/dl)	1.5 ± 0.8	1.3 ± 0.5	0.230
Bilirubin (mg/dl)	2.6 ± 1.9	2.5 ± 1.3	0.576
INR	1.2 ± 1.9	1.3 ± 2.2	0.280
Albumin (g/dl)	3.5 ± 0.4	2.4 ± 0.4	**<0.001**
Postoperative characteristics			
ECMO	33 (19.2)	28 (50)	**<0.001**
Renal replacement therapy	90 (57.7)	35 (62.5)	0.635
MELD score	14.6 ± 5.3	14.3 ± 5.1	0.711
Albumin substitution within first 24 h	47 (28.7)	29 (54.7)	**0.001**
Days in ICU	24 ± 25	25 ± 26	0.677
Duration of mechanical ventilation (hours)	110 ± 167	223 ± 216	**0.001**
30-day mortality	6 (3.5)	12 (21.4)	**<0.001**
1-year mortality	18 (10.4)	22 (39.3)	**<0.001**

ARVC, arrhythmogenic right ventricular cardiomyopathy; BMI, body mass index; CPB, cardiopulmonary bypass; DCM, dilative cardiomyopathy; ECMO; extracorporeal membrane oxygenation; FFP, fresh frozen plasma; HCM, hypertrophic cardiomyopathy; ICM, ischaemic cardiomyopathy; IMPACT, risk index for mortality prediction after cardiac transplantation; ICU, intensive care unit; INR, international normalized ratio; LVAD, left ventricular assist device; MELD, model for end-stage liver disease; PRBC, packed red blood cells; RCM, restrictive cardiomyopathy [11].

Significant results are marked in bold.

### Pre- and postoperative serum albumin levels in survivors and non-survivors

**Figure 1:**
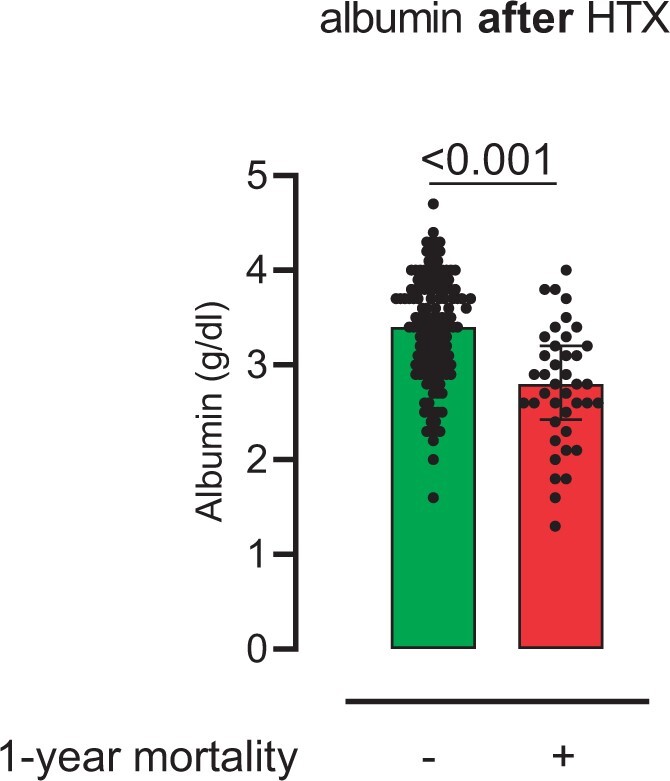
Postoperative serum albumin levels. The figure shows postoperative serum albumin levels after heart transplantation in survivors compared with non-survivors 1-year follow-up (survivors: 3.3 ± 0.6 g/dl vs non-survivors: 2.8 ± 0.6 g/dl; *P* < 0.001)

There was no significant difference in preoperative serum albumin levels between survivors and non-survivors (survivors: 3.9 ± 0.7 g/dl vs non-survivors: 3.7 ± 0.7 g/dl, *P* = 0.084). Postoperative serum albumin levels were significantly lower in patients who died in the first year after HTX (survivors: 3.3 ± 0.6 g/dl vs non-survivors: 2.8 ± 0.6 g/dl, *P* < 0.001) (Fig. 1).

### Discrimination and association of postoperative albumin and 1-year mortality

**Figure 2:**
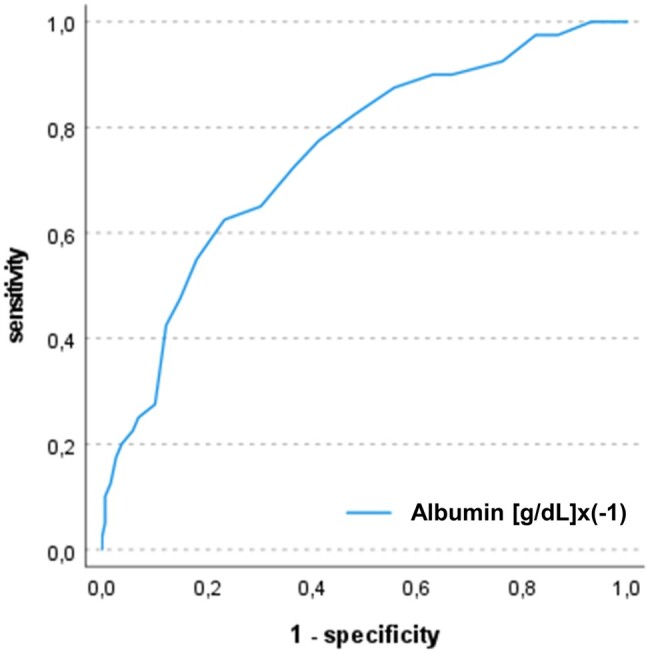
Receiver operating characteristic curves of postoperative albumin levels and 1-year mortality. The figure shows the ROC curves for association of postoperative albumin levels with 1-year mortality after heart transplantation. The AUC was 0.75 (95% CI: 0.66–0.83)

**Figure 3:**
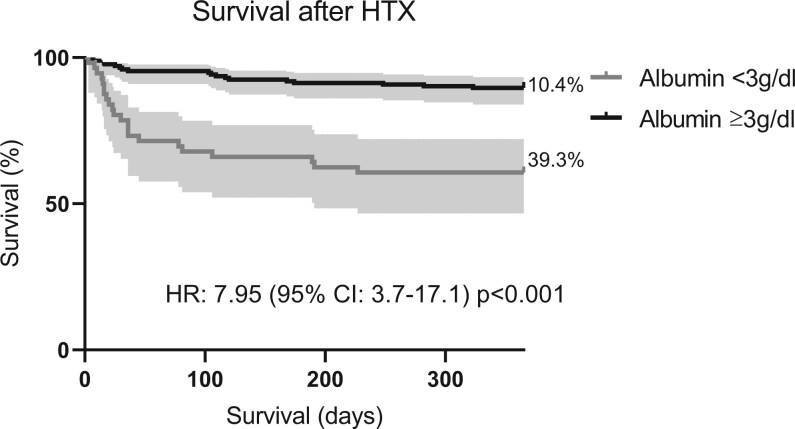
Postoperative survival of patients based on albumin levels. The figure shows the Kaplan–Meier curves of patients after HTX classified by postoperative albumin levels above and below cutoff of 3 g/dl. Survival in patients with postoperative albumin levels >3 g/dl was higher as compared to controls (HR 7.95, 95% CI: 3.7–17.1; *P*<0.001)

In ROC analysis, postoperative albumin levels showed a significant discrimination for 1-year mortality with an area under the curve (AUC) of 0.75 and 95% CI of 0.66–0.83. Youden index determined a cutoff of 2.95 g/dl for postoperative albumin levels. In a univariate logistic regression model, there was a significant association between postoperative serum albumin values and 1-year mortality (OR: 4.54, 95% CI: 2.34–8.78, *P*≤0.001). In Kaplan–Meier analysis, patients with postoperative albumin levels below cutoff showed lower survival rates as compared to controls (hazard ratio 7.95, 95% CI: 3.7–17.1, *P* < 0.001). Of note, preoperative hypoalbuminaemia occurred in 19 patients (8%) and was not associated with the incidence of postoperative hypoalbuminaemia. After adjustment for 13 covariables in multivariate logistic regression analysis, only postoperative albumin and donor age remained independently associated with 1-year mortality (postoperative albumin—OR: 4.76, 95% CI: 1.94–11.67, *P* = 0.001 and donor age—OR: 1.11, 95% CI: 1.05–1.17, *P≤*0.001] (Figs 2 and 3, Table 2).

**Table 2:** Multivariate binary logistic regression for the association of postoperative albumin and 1-year mortality

Parameter	Adjusted odds ratio	95% CI	P-value
Albumin (g/dl) × (−1)	4.76	1.94–11.67	**0.001**
IMPACT	1.04	0.91–1.19	0.549
Postoperative MELD	1.05	0.94–1.17	0.424
Recipient diabetes	1.09	0.31–3.78	0.892
Prior LVAD	0.86	0.20–3.77	0.838
Prior cardiothoracic surgery	1.50	0.31–7.33	0.618
Donor age (years)	1.11	1.05–1.17	**<0.001**
Duration of surgery (min)	0.99	0.98–0.99	**0.015**
Duration of CPB (min)	1.01	0.99–1.02	0.221
mechanical ventilation (h)	1.00	1.00–1.01	0.073
Postoperative RRT	1.78	0.47–6.68	0.396
Postoperative ECMO	1.96	0.54–7.07	0.305
PRBC transfusion (ml)	1.00	1.00–1.00	0.774
Platelet transfusion (ml)	1.00	1.00–1.00	0.154

CPB, cardiopulmonary bypass; ECMO, extracorporeal membrane oxygenation; IMPACT, risk index for mortality prediction after cardiac transplantation; LVAD, left ventricular assist device; MELD, model for end-stage liver disease; PRBC, packed red blood cells; RRT, renal replacement therapy [11].

### Improvement of risk prediction models by postoperative albumin

**Figure 4:**
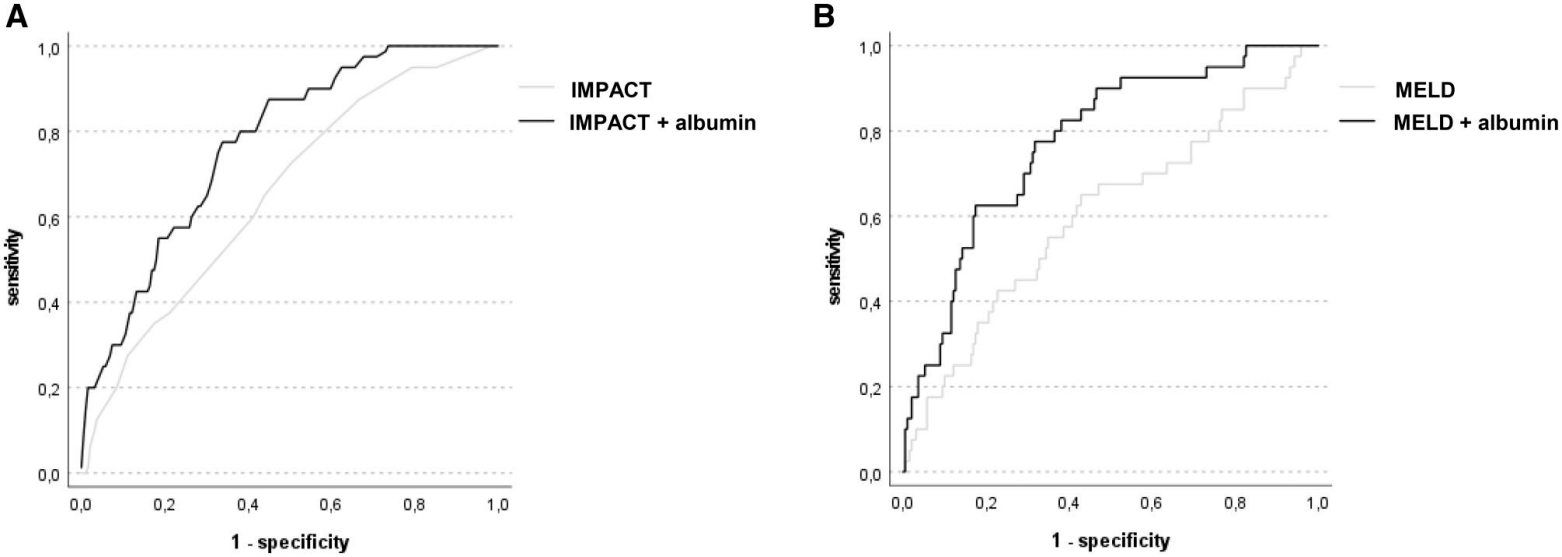
Receiver operating characteristic curves of two different prediction models for 1-year mortality including postoperative albumin. ROC curves using the IMPACT score (**A**) or the MELD score (**B**) alone compared to models which added postoperative albumin levels (dependent variable: 1-year mortality). Models including postoperative albumin showed better discrimination for 1-year mortality (IMPACT score—AUC = 0.65, 95% CI: 0.57–0.74 vs IMPACT score with albumin—AUC = 0.77, 95% CI: 0.70–0.84; difference between areas: 0.12, 95% CI: 0.02–0.21, *P* = 0.016; MELD score—AUC = 0.60, 95% CI: 0.50–0.70 vs. MELD score with albumin—AUC = 0.78, 95% CI: 0.70–0.85; difference between areas: 0.17, 95% CI: 0.06–0.29, *P* = 0.002)

We analysed if risk prediction of either IMPACT or MELD score for 1-year mortality could be improved by the addition of postoperative albumin levels to the prognostic models (based on logistic regression). The NRI for the model including IMPACT and albumin was 15.87% (95% CI: 9.43–23.53) for non-events and 5% (95% CI: 1.64–11.28) for events. The NRI for the model including MELD and albumin was 4.23% (95% CI: 1.64–9.93) for non-events and 25% (95% CI: 16.88–34.66) for events. The assessment of NARI showed that both models including postoperative albumin were able to detect 139/1000 and 78/1000 patients more at risk for 1-year mortality, respectively. ROC analysis showed that the models including postoperative albumin levels had significantly higher AUC as compared to the two baseline models only including IMPACT score (IMPACT score—AUC = 0.65, 95% CI: 0.57–0.74; IMPACT score with albumin—AUC = 0.77, 95% CI: 0.70–0.84; difference between areas: 0.12, 95% CI: 0.02–0.21, *P* = 0.016) or MELD score (MELD score—AUC = 0.60, 95% CI: 0.50–0.70; MELD score with albumin—AUC = 0.78, 95% CI: 0.70–0.85; difference between areas: 0.17, 95% CI: 0.06–0.29, *P* = 0.002) (Fig. 4, Supplementary Tables S2 and S3).

### Association of postoperative albumin with DAOH

In univariate analysis, DAOH of patients with postoperative albumin levels ≥3 g/dl were significantly higher than DAOH of patients with albumin levels <3 g/dl [albumin—above cut-off 308 (271–325) days vs below cutoff 253 (0–305) days, *P ≤ *0.001]. Of note, after the exclusion of patients who died during 1-year follow-up, univariate findings for DAOH remained significant in a sensitivity analysis (Supplementary Figure S2). A multivariate quantile regression model was performed in which postoperative albumin levels were independently associated with poor DAOH after adjustment for 13 covariables (postoperative albumin—coefficient: −46.97, 95% CI: −83.81 to −10.13, *P* = 0.013) (Table 3, Supplementary Figure S3).

**Table 3:** Multivariate quantile regression model for the association of postoperative albumin with Days alive and out of hospital

Parameter	Coefficient	Standard error	95% CI	P-value
Constant	163.39	95.20	−24.49 to 351.27	0.088
Albumin (g/dl) × (−1)	−46.97	18.67	−83.81 to −10.13	**0.013**
MELD	−0.09	0.39	−0.86–0.68	0.821
IMPACT	−3.42	2.67	−8.70 to 1.85	0.202
Recipient diabetes	−73.40	25.26	−123.24 to −23.56	**0.004**
Prior LVAD	13.75	30.82	−47.07 to 74.58	0.656
Prior cardiothoracic surgery	18.73	31.40	−43.25 to 80.70	0.552
Donor age (years)	−2.80	0.81	−4.40 to −1.20	**<0.001**
Duration of surgery (min)	0.08	0.15	−0.21 to 0.37	0.607
Duration of CPB (min)	0.21	0.23	−0.24 to 0.67	0.355
mechanical ventilation (h)	−0.14	0.07	−0.28 to 0.01	0.056
Postoperative RRT	−120.91	22.59	−165.49 to −76.34	**<0.001**
Postoperative ECMO	−3.60	29.22	−61.26 to 54.05	0.902
PRBC transfusion (ml)	−0.016	0.01	−0.03 to −0.01	**0.006**
Platelet transfusion (ml)	0.01	0.02	−0.02 to 0.05	0.405

CPB, cardiopulmonary bypass; ECMO, extracorporeal membrane oxygenation; IMPACT, risk index for mortality prediction after cardiac transplantation; LVAD, left ventricular assist device; MELD, model for end-stage liver disease; PRBC, packed red blood cells; RRT, renal replacement therapy [11].

## DISCUSSION

This study revealed an independent association between low postoperative serum albumin levels and increased 1-year mortality as well as poor DAOH after HTX. Additionally, risk prediction for mortality by IMPACT score or MELD score was significantly improved when postoperative albumin was added to the models.

### The role of preoperative serum albumin in patients undergoing HTX

Previous investigations described that preoperative serum albumin levels were associated with unfavourable outcome after HTX. Kato *et al.* [8] showed in a retrospective analysis of 822 HTX patients that preoperative serum albumin levels <3.5 g/dl were associated with increased 1-year mortality. Additionally, the authors used the same cutoff for analysis of data of 13 671 HTX patients from the united network of organ sharing database. Again preoperative serum albumin levels below cutoff were associated with poor 1-year survival in a parametric survival model [15]. Other studies showed similar associations between preoperative albumin levels and 1-year mortality after HTX [6]. Previously preoperative albumin levels were also included in risk scores to assess postoperative survival after HTX. In this context, Schulze *et al.* [16] proposed the CARRS score including prior stroke, albumin, retransplantation, glomerular filtration rate and prior thoracic surgeries. This score showed a good discriminative ability with an ROC-AUC of 0.77. Another study by Chokshi *et al.* [7] investigated the role of liver dysfunction on outcome after HTX. Therefore, preoperative serum albumin values were added to the MELD score. Higher modified MELD score and lower albumin values were associated with poor survival.

### The role of postoperative serum albumin after HTX

The role of postoperative serum albumin levels after HTX has not been investigated until now. Our current data suggest that low postoperative serum albumin values are strongly associated with poor outcome regarding 1-year survival and DAOH after HTX. This is in line with the findings of previous studies for cardiac and non-cardiac surgery [2, 17]. Berbel-Franco *et al.* [1] showed a strong association of low postoperative serum albumin levels (within the first 24 h at ICU) with in-hospital and long-term mortality in 2818 cardiac surgery patients. Interestingly risk for mortality was not linear but showed a progressively steeper increase when serum albumin levels were below 3.0 g/dl which goes in line with the cutoff from our ROC analysis [1]. However, heart transplant patients were not included in the above-mentioned study. It is well known that postoperative serum albumin mimics intra- and postoperative course as it is influenced by numerous factors like increased inflammatory response resulting from surgical trauma, long CPB times or ischaemia reperfusion injury and postoperative liver dysfunction, making it a suitable biomarker for postoperative prognosis [1, 2, 18–20]. Within our patient cohort, we could show that patients with serum albumin levels below the cutoff of 3 g/dl had significantly longer durations of surgery and CPB, as well as significantly higher transfusion requirements, supporting this relationship. This might be the reason why postoperative albumin showed a strong independent association with mortality and DAOH in our cohort. Additionally, we adjusted our findings for postoperative MELD score, which shows that low albumin values are not solely influenced by postoperative liver dysfunction and impaired synthesis after HTX. Although, preoperative factors are more useful for risk prediction than postoperative variables, postoperative albumin can be used for early risk re-assessment after HTX, as a widely available biomarker in clinical practice. If low serum albumin levels themselves have negative effects on patient’s prognosis after HTX is unclear, but could be an interesting therapeutic approach for upcoming investigations. Targeting postoperative serum albumin levels >3.0 g/dl after HTX by substitution of human albumin might be a feasible approach and should be investigated in prospective trials, as data concerning this topic are lacking. However, as in our cohort patients with low albumin levels had higher rates of albumin substitution but also higher mortality, benefit of this therapeutic approach is questionable and cannot be drawn from our data, as negative effects should also be considered.

### Strengths and limitations

This work has several limitations that we are aware of. First, this was a retrospective single-centre study with a limited number of patients and events. However, data were obtained from the local HTX database and we had a full dataset regarding the endpoints and covariates in 95% of all patients. As our patients are closely connected to our centre we could provide not only data on mortality but also DAOH which represents a more patient-centred outcome. Nevertheless, we cannot be sure if patients were hospitalized externally during 1-year follow-up, although the risk is very limited. Finally, we had to assess the postoperative albumin values during the first 12 h after arrival ICU, as albumin was not measured systematically at arrival in ICU in all patients. Regarding our statistical approach, all analyses were exploratory in nature and 95% CIs were not adjusted for multiple comparisons, hence inferences drawn from them may not be reproducible.

## CONCLUSIONS

Early postoperative hypoalbuminaemia <3.0 g/dl is associated with 1-year mortality and poor DAOH after HTX. This makes postoperative albumin a suitable marker for early risk re-assessment after HTX.

## Supplementary Material

ivae012_Supplementary_Data

## Data Availability

All relevant data are included in the present manuscript or in the supplements. Raw data are available upon reasonable request by the first author R.M.

## Notes

Abstracts including data from the same data set have been submitted to the annual meeting 2023 of the European Association for Cardio-Thoracic Surgery (EACTS; no presentation) and to the annual meeting 2023 of the European Society of Anaesthesiology and Intensive Care (ESAIC; oral presentation).

## References

[1] Berbel-Franco D , Lopez-Delgado JC, Putzu A, Esteve F, Torrado H, Farrero E et al The influence of postoperative albumin levels on the outcome of cardiac surgery. J Cardiothorac Surg 2020;15:78. 32393356 10.1186/s13019-020-01133-y PMC7216430

[2] Lee EH , Chin JH, Choi DK, Hwang BY, Choo SJ, Song JG et al Postoperative hypoalbuminemia is associated with outcome in patients undergoing off-pump coronary artery bypass graft surgery. J Cardiothorac Vasc Anesth 2011;25:462–8. 21093290 10.1053/j.jvca.2010.09.008

[3] Kilic A , Allen JG, Weiss ES. Validation of the United States-derived Index for Mortality Prediction After Cardiac Transplantation (IMPACT) using international registry data. J Heart Lung Transplant 2013;32:492–8. 23474362 10.1016/j.healun.2013.02.001

[4] Weiss ES , Allen JG, Arnaoutakis GJ, George TJ, Russell SD, Shah AS et al Creation of a quantitative recipient risk index for mortality prediction after cardiac transplantation (IMPACT). Ann Thorac Surg 2011;92:914–21; discussion 921–2. 21871277 10.1016/j.athoracsur.2011.04.030

[5] Loforte A , Fiorentino M, Gliozzi G, Mariani C, Folesani G, Suarez SM et al Heart transplant and hepato-renal dysfunction: the model of end-stage liver disease excluding international normalized ratio as a predictor of postoperative outcomes. Transplant Proc 2019;51:2962–6. 31607616 10.1016/j.transproceed.2019.07.013

[6] Zhou Y , Chen S, Rao Z, Yang D, Liu X, Dong N et al Prediction of 1-year mortality after heart transplantation using machine learning approaches: a single-center study from China. Int J Cardiol 2021;339:21–7. 34271025 10.1016/j.ijcard.2021.07.024

[7] Chokshi A , Cheema FH, Schaefle KJ, Jiang J, Collado E, Shahzad K et al Hepatic dysfunction and survival after orthotopic heart transplantation: application of the MELD scoring system for outcome prediction. J Heart Lung Transplant 2012;31:591–600. 22458996 10.1016/j.healun.2012.02.008 PMC3358427

[8] Kato TS , Cheema FH, Yang J, Kawano Y, Takayama H, Naka Y et al Preoperative serum albumin levels predict 1-year postoperative survival of patients undergoing heart transplantation. Circ Heart Fail 2013;6:785–91. 23674361 10.1161/CIRCHEARTFAILURE.111.000358 PMC4074373

[9] Liao S , Lu X, Cheang I, Zhu X, Yin T, Yao W et al Prognostic value of the modified model for end-stage liver disease (MELD) score including albumin in acute heart failure. BMC Cardiovasc Disord 2021;21:128. 33750318 10.1186/s12872-021-01941-7 PMC7941696

[10] Ghaferi AA , Schwartz TA, Pawlik TM. STROBE reporting guidelines for observational studies. JAMA Surg 2021;156:577–8. 33825815 10.1001/jamasurg.2021.0528

[11] M'Pembele R , Roth S, Nucaro A, Stroda A, Tenge T, Lurati Buse G et al Postoperative high-sensitivity troponin T predicts 1-year mortality and days alive and out of hospital after orthotopic heart transplantation. Eur J Med Res 2023;28:16. 36624515 10.1186/s40001-022-00978-4 PMC9827673

[12] Kamath PS , Wiesner RH, Malinchoc M, Kremers W, Therneau TM, Kosberg CL et al A model to predict survival in patients with end-stage liver disease. Hepatology 2001;33:464–70. 11172350 10.1053/jhep.2001.22172

[13] Ariti CA , Cleland JG, Pocock SJ, Pfeffer MA, Swedberg K, Granger CB et al Days alive and out of hospital and the patient journey in patients with heart failure: insights from the candesartan in heart failure: assessment of reduction in mortality and morbidity (CHARM) program. Am Heart J 2011;162:900–6. 22093207 10.1016/j.ahj.2011.08.003

[14] M'Pembele R , Roth S, Stroda A, Reier T, Lurati Buse G, Sixt SU et al Validation of days alive and out of hospital as a new patient-centered outcome to quantify life impact after heart transplantation. Sci Rep 2022;12:18352. 36319821 10.1038/s41598-022-21936-4 PMC9626454

[15] Kato TS , Lippel M, Naka Y, Mancini DM, Schulze PC. Post-transplant survival estimation using pre-operative albumin levels. J Heart Lung Transplant 2014;33:547–8. 24656284 10.1016/j.healun.2014.01.921 PMC4039657

[16] Schulze PC , Jiang J, Yang J, Cheema FH, Schaeffle K, Kato TS et al Preoperative assessment of high-risk candidates to predict survival after heart transplantation. Circ Heart Fail 2013;6:527–34. 23505300 10.1161/CIRCHEARTFAILURE.112.000092 PMC4283202

[17] Labgaa I , Joliat GR, Kefleyesus A, Mantziari S, Schafer M, Demartines N et al Is postoperative decrease of serum albumin an early predictor of complications after major abdominal surgery? A prospective cohort study in a European centre. BMJ Open 2017;7:e013966. 10.1136/bmjopen-2016-013966 PMC5775466 28391235

[18] Hübner M , Mantziari S, Demartines N, Pralong F, Coti-Bertrand P, Schäfer M. Postoperative albumin drop is a marker for surgical stress and a predictor for clinical outcome: a pilot study. Gastroenterol Res Pract 2016;2016:8743187. 26880899 10.1155/2016/8743187 PMC4736779

[19] Mantziari S , Hübner M, Coti-Bertrand P, Pralong F, Demartines N, Schäfer M. *A* Novel approach to major surgery: tracking its pathophysiologic footprints. World J Surg 2015;39:2641–51. 26243563 10.1007/s00268-015-3181-7

[20] Corral-Velez V , Lopez-Delgado JC, Betancur-Zambrano NL, Lopez-Suñe N, Rojas-Lora M, Torrado H et al The inflammatory response in cardiac surgery: an overview of the pathophysiology and clinical implications. Inflamm Allergy Drug Targets 2015;13:367–70. 26021321 10.2174/1871528114666150529120801

