# Supplementary material for: Association between early postoperative hypoalbuminaemia and outcome after orthotopic heart transplantation
Source: Interdiscip Cardiovasc Thorac Surg. 2024 Jan 16;38(1):ivae012. doi: 10.1093/icvts/ivae012 (PMC10827358; doi:10.1093/icvts/ivae012)
Supplement: ivae012_Supplementary_Data [file ivae012_supplementary_data.docx]

**Supplementary material:**

**Figure S1: Study flowchart**

**
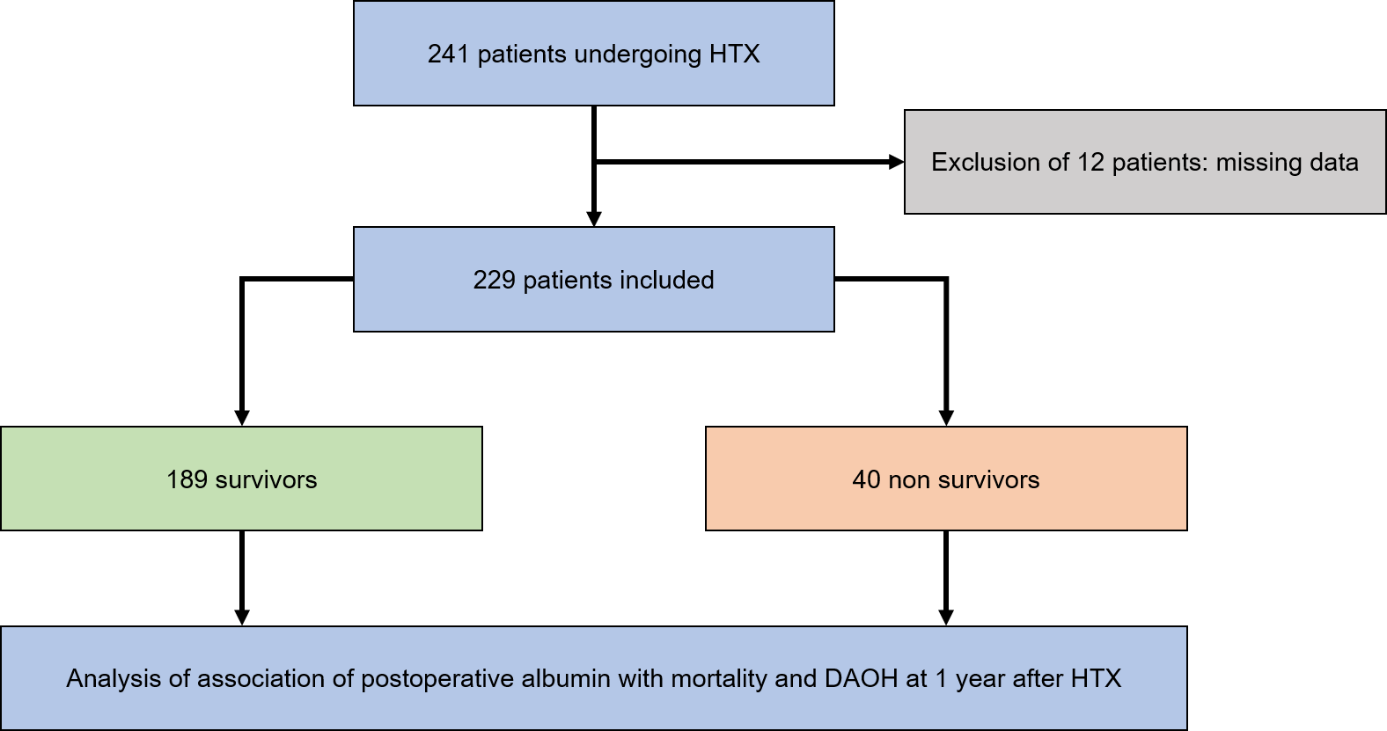
**

**Figure S2: Days out of hospital depending on postoperative serum albumin cutoff**

**
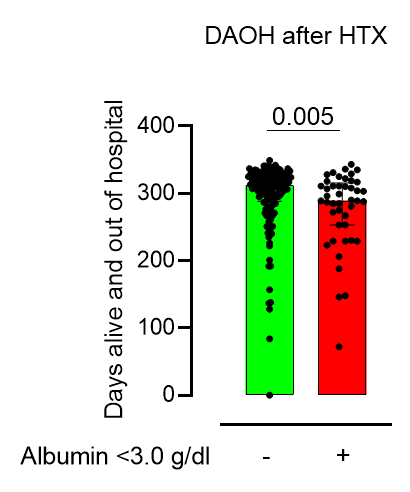
**

**Legend:** The figure shows days out of hospital of patients with postoperative albumin above and below the cutoff of 3.0 g/dl. For sensitivity analysis, all patients who died within the first year after HTX were excluded. Patients with albumin below cutoff had significantly lower days out of hospital [Albumin >3.0g/dl - 312 (289-327) days vs. Albumin <3.0 g/dl 290 (253 -316) days, p = 0.005].

**Figure S3: Multivariate quantile regression model for association of postoperative albumin with Days alive and out of hospital.**


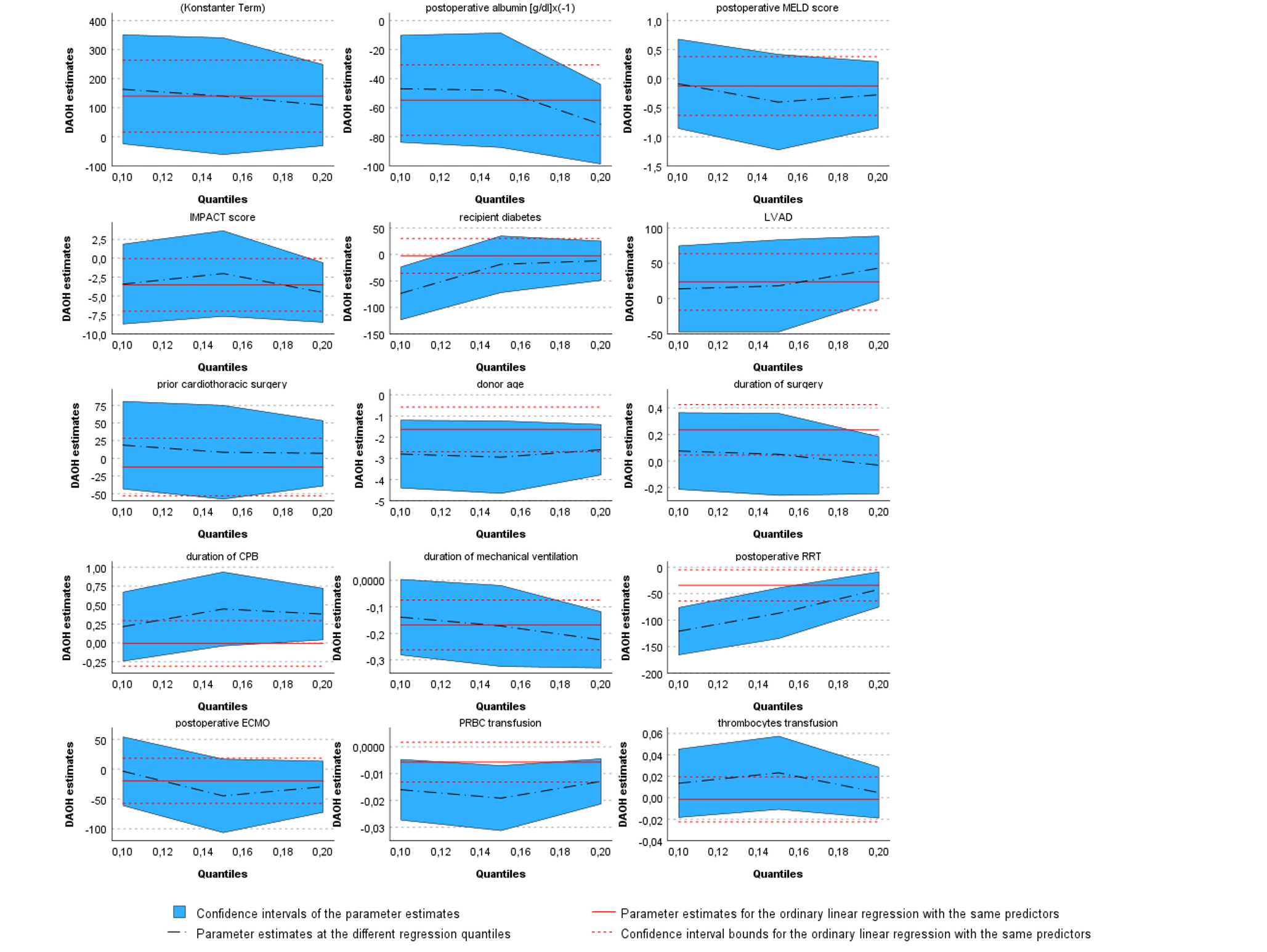


**Legend:** The figure shows a quantile regression model for the association of postoperative albumin with DAOH adjusted for various covariables. The Regression was conducted for the two lowest centiles of DAOH.

**Tables:**

**Table S1: Patient characteristics of survivors versus non-survivors**

|  | **Survivors**  **(N=189)** | **Non-survivors**  **(N=40)** | **p-value^a^** |  |
| --- | --- | --- | --- | --- |
| **Preoperative**  **recipient**  **characteristics** |  |  |  |  |
| male sex | 141 (74.6) | 26 (65) | 0.241 |  |
| Age (years) | 54.8 ± 10.8 | 57.2 ± 11.1 | 0.194 |  |
| BMI (kg/m²) | 25.6 ± 4.4 | 25.9 ± 5.1 | 0.654 |  |
| smoker | 47 (25) | 10 (25) | >0.999 |  |
| diabetes | 39 (20.9) | 11 (27.5) | 0.401 |  |
| arterial hypertension | 109 (58) | 21 (52.5) | 0.599 |  |
| pulmonary hypertension | 16 (8.5) | 5 (12.5) | 0.382 |  |
| prior cardiothoracic surgery | 116 (61.4) | 29 (72.5) | 0.210 |  |
| LVAD | 92 (48.7) | 23 (57.5) | 0.385 |  |
| ICM | 80 (42.6) | 18 (45) | 0.861 |  |
| DCM | 92 (48.9) | 17 (42.5) | 0.490 |  |
| ARVC | 6 (3.2) | 2 (5) | 0.632 |  |
| RCM | 0 (0) | 1 (2.5) | 0.175 |  |
| HCM | 3 (1.6) | 1 (2.5) | 0.540 |  |
| myocarditis | 2 (1.1) | 0 (0) | >0.999 |  |
| preoperative dialysis | 9 (4.8) | 3 (7.7) | 0.439 |  |
| IMPACT score | 8.2 ± 4.6 | 10.4 ± 4.4 | **0.003** |  |
| albumin (g/dL) | 3.9 ± 0.7 | 3.7 ± 0.7 | 0.084 |  |
| MELD-score | 13.5 ± 7 | 16.9 ± 8 | **0.018** |  |
| **Donor characteristics** |  |  |  |  |
| Male sex | 109 (57.7) | 17 (42.5) | 0.084 |  |
| Age (years) | 42.1 ± 12.4 | 49.6 ± 9.6 | **<0.001** |  |
| BMI (kg/m²) | 26.1 ± 4.8 | 25.6 ± 3.6 | 0.505 |  |
| Cardiopulmonary resuscitation | 60 (31.7) | 8 (20) | 0.182 |  |
| **Intraoperative characteristics (min)** |  |  |  |  |
| duration of surgery | 417 ± 105 | 452 ± 158 | 0.186 |  |
| duration of CPB | 247 ± 63 | 285 ± 99 | **0.002** |  |
| total ischemia time | 212 ± 48 | 226 ± 51 | 0.097 |  |
| PRBC (L) | 2.9 ± 2.2 | 5.3 ± 3.9 | **0.001** |  |
| Platelets (L) | 1.0 ± 0.7 | 1.6 ± 1.2 | **0.008** |  |
| FFP (L) | 1.4 ± 1.5 | 2.5 ± 2.6 | **0.013** |  |
| **Postoperative laboratory values** |  |  |  |  |
| creatinine (mg/dl) | 1.4 ± 0.8 | 1.4 ± 0.5 | 0.815 |  |
| bilirubin (mg/dl) | 2.5 ± 1.7 | 3.1 ± 1.7 | 0.082 |  |
| INR | 1.2 ± 0.2 | 1.3 ± 0.2 | **0.012** |  |
| albumin (g/dL) | 3.3 ± 0.6 | 2.8 ± 0.6 | **<0.001** |  |
| **Postoperative characteristics** |  |  |  |  |
| ECMO | 39 (20.7) | 22 (55) | **<0.001** |  |
| renal replacement therapy | 94 (54) | 31 (81.6) | **0.002** |  |
| MELD-score | 14.1 ± 5 | 16.1 ± 5.8 | **0.034** |  |
| Albumin substitution within first 24 hours | 57 (31.8) | 19 (50) | **0.040** |  |
| days in ICU | 22.3 ± 21.8 | 31.5 ± 37.2 | **0.038** |  |
| duration of mechanical ventilation (hours) | 112 ± 164 | 274 ± 234 | **<0.001** |  |
| 30-day mortality | 0 (0) | 18 (45) | **<0.001** |  |

**Table S2: Reclassification tables of a 1-year mortality prediction model using IMPACT compared to a model using IMPACT and postoperative albumin.**

**A**

| **Reclassification table non-events** | | | | | |
| --- | --- | --- | --- | --- | --- |
| **Model** |  | IMPACT + postoperative albumin | | | Total |
|  | **Riskgroup** | low | intermediate | high |  |
| IMPACT | Low | 41 | 16 | 6 | 63 |
|  | intermediate | 17 | 21 | 10 | 48 |
|  | high | 13 | 31 | 34 | 78 |
| Total | | 71 | 68 | 50 | 189 |

**B**

| **Reclassification tabel events** | | | | | |
| --- | --- | --- | --- | --- | --- |
| **Model** |  | IMPACT + postoperative albumin | | | Total |
|  | **Risk group** | low | intermediate | high |  |
| IMPACT | low | 1 | 2 | 2 | 5 |
|  | intermediate | 0 | 5 | 6 | 11 |
|  | high | 1 | 7 | 16 | 24 |
| Total | | 2 | 14 | 24 | 40 |

**Legend:** Tables show reclassification of risk for 1-year mortality in patients undergoing HTX by a model using IMPACT combined with albumin compared to a model using IMPACT alone. Table A shows reclassification in 189 patients who did not experience 1-year mortality (non-events). The new model including albumin correctly downrated the risk of 62 patients (true negative) while falsely uprated the risk of 32 patients (false positive). Table B shows reclassification in 40 patients who died within the first year after HTX (events). The new model including albumin correctly uprated the risk of 10 patients (true positive) while falsely downrated the risk of 8 patients (false negative).

**Table S3: Reclassification tables of a 1-year mortality prediction model using MELD compared to a model using MELD and postoperative albumin.**

**A**

| **Reclassification table non-events** | | | | | |
| --- | --- | --- | --- | --- | --- |
| **Model** |  | MELD + postoperative albumin | | | Total |
|  | **Riskgroup** | low | intermediate | high |  |
| MELD | Low | 32 | 26 | 8 | 66 |
|  | intermediate | 28 | 22 | 16 | 66 |
|  | high | 13 | 17 | 27 | 57 |
| Total | | 73 | 65 | 51 | 189 |

**B**

| **Reclassification tabel events** | | | | | |
| --- | --- | --- | --- | --- | --- |
| **Model** |  | MELD + postoperative albumin | | | Total |
|  | **Risk group** | low | intermediate | high |  |
| MELD | low | 1 | 4 | 6 | 11 |
|  | intermediate | 1 | 4 | 6 | 11 |
|  | high | 1 | 4 | 13 | 18 |
| Total | | 3 | 12 | 25 | 40 |

**Legend:** Tables show reclassification of risk for 1-year mortality in patients undergoing HTX by a model using MELD combined with albumin compared to a model using MELD alone. Table A shows reclassification in 189 patients who did not experience 1-year mortality (non-events). The new model including albumin correctly downrated the risk of 58 patients (true negative) while falsely uprated the risk of 50 patients (false positive). Table B shows reclassification in 40 patients who died within the first year after HTX (events). The new model including albumin correctly uprated the risk of 16 patients (true positive) while falsely downrated the risk of 6 patients (false negative).
